# Ensuring competency in focused cardiac ultrasound: a systematic review of training programs

**DOI:** 10.1186/s40560-020-00503-x

**Published:** 2020-12-11

**Authors:** Lauren E. Gibson, Gabrielle A. White-Dzuro, Patrick J. Lindsay, Sheri M. Berg, Edward A. Bittner, Marvin G. Chang

**Affiliations:** Department of Anesthesia, Critical Care, and Pain Medicine, Massachusetts General Hospital, 55 Fruit Street GRB 444, Boston, MA 02114 USA

**Keywords:** Cardiac ultrasound, Point-of-care ultrasound, Bedside ultrasound, Ultrasonography, Training, Education, Assessment

## Abstract

**Background:**

Focused cardiac ultrasound (FoCUS) is a valuable skill for rapid assessment of cardiac function and volume status. Despite recent widespread adoption among physicians, there is limited data on the optimal training methods for teaching FoCUS and metrics for determining competency. We conducted a systematic review to gain insight on the optimal training strategies, including type and duration, that would allow physicians to achieve basic competency in FoCUS.

**Methods:**

Embase, PubMed, and Cochrane Library databases were searched from inception to June 2020. Included studies described standardized training programs for at least 5 medical students or physicians on adult FoCUS, followed by an assessment of competency relative to an expert. Data were extracted, and bias was assessed for each study.

**Results:**

Data were extracted from 23 studies on 292 learners. Existing FoCUS training programs remain varied in duration and type of training. Learners achieved near perfect agreement (*κ* > 0.8) with expert echocardiographers on detecting left ventricular systolic dysfunction and pericardial effusion with 6 h each of didactics and hands-on training. Substantial agreement (*κ* > 0.6) on could be achieved in half this time.

**Conclusion:**

A short training program will allow most learners to achieve competency in detecting left ventricular systolic dysfunction and pericardial effusion by FoCUS. Additional training is necessary to ensure skill retention, improve efficiency in image acquisition, and detect other pathologies.

## Background

Technological advancements have led to increasing availability of high quality, low-profile ultrasound devices at reduced costs [1]. One area that has seen tremendous growth is that of focused cardiac ultrasound (FoCUS), which describes point-of-care ultrasound that is intended to provide a rapid qualitative assessment of cardiac function. The use of FoCUS has expanded to a variety of practice settings, including emergency medicine, critical care, anesthesia, internal medicine, and primary care, owing largely to its relative ease of use [2]. Prior studies suggest that trainees and non-cardiologist physicians with limited prior ultrasonographic experience can gain proficiency in FoCUS with brief training, such as a 1-day workshop and 20–50 practice scans [3, 4]. FoCUS has proven useful for the assessment of ventricular function, valvular abnormalities, volume status, as well as for the detection of cardiac tamponade, aortic dissection or aneurysm, and pulmonary embolism [5]. The use of FoCUS has been shown to alter management in perioperative [6, 7], critical care [8, 9], and emergency [10, 11] settings and has been shown to improve outcomes in select patients [12].

While FoCUS can be beneficial for patient care and more effective allocation of healthcare resources, there is potential for harm with inappropriate use [13]. The implications of relying on a false negative exam could include delayed or missed diagnoses. Similarly, false positive findings or misinterpretations could lead to unwarranted testing or procedures and increased healthcare spending. Despite the potential for such consequences, formal training programs have not been widely embraced, and quality control metrics are often lacking [14, 15]. Surveys have revealed the fear of missed diagnoses and the lack of training or certification as important barriers to the adoption of FoCUS [16]. The adoption of robust parameters for assessing competency in image acquisition, analysis, and interpretation among physicians is needed to effectively train learners and ensure appropriate use [17].

Leaders in ultrasonography have recognized the need for training standards and have supported the development of structured certification programs for FoCUS as well as quantitative transthoracic echocardiography (TTE), shown in Table 1 [24, 31]. Current certifications in TTE require between 75 and 250 scans and passing one or more standardized examinations, while certification in FoCUS typically requires between 20 and 50 supervised scans. However, many of these recommendations are based on guidance developed for the use of FoCUS and/or TTE in emergency and critical care settings, and their applicability outside of these settings has not been well-demonstrated. There is also no consensus on the optimal method of training in FoCUS or the appropriate metrics for determining skill development. Many small-scale studies have documented and compared strategies for FoCUS education and evaluation among various sub-populations and clinical environments [32]. Among these are studies on trainees and licensed physicians working in intensive care units, medical wards, emergency departments, and perioperative areas for which very different scanning protocols are employed. The heterogeneity of studies has made it difficult to draw conclusions, and thus, the type and duration of training to allow most learners to achieve competency in FoCUS remains undetermined. We conducted a systematic review and meta-analysis to examine existing strategies for FoCUS training and to gain insight on the optimal amount and type of training that will allow for attainment of basic competency in adult FoCUS.

**Table 1:** Published accreditations in focused cardiac ultrasound and transthoracic echocardiography

Organization(s)	Applicable to	Recommendations/requirements
**Accreditations in focused cardiac ultrasound**		
American College of Chest Physicians [18], Society of Hospital Medicine [19]	US internal medicine and family medicine physicians	**Point-of-care ultrasound certificate of completion**—attend a 2-day course, completion of an online learning module, submission of 20 focused exams, final examination
American College of Emergency Physicians [20]	US emergency medicine physicians	Completion of a dedicated ultrasound course or a 1–2 week preceptorship, followed by a period of supervision during clinical application; recommend completion of 25–50 exams in each domain
Society of Point-of-Care Ultrasound [21]	US physicians, nurses, EMS personnel	Recommend 25–50 supervised exams for each domain (with 5% demonstrating pathology), followed by ongoing quality assurance
Society of Critical Care Medicine [22]	US critical care physicians	At least 20 didactic hours, must perform 30 and interpret 50 focused exams
British Society of Echocardiography, Intensive Care Society [23]	UK critical care physicians	Attend an approved 1-day course, perform 50 exams (at least 10 fully supervised), submit log of exam reports for review by a certified mentor
European Society of Intensive Care Medicine [24]	European critical care physicians	Recommend 10 h of combined didactic and practical training and completion of 30 supervised exams
**Accreditations in transthoracic echocardiography**		
American College of Cardiology, American Society of Echocardiography [25]	US cardiology trainees to obtain early level competency	**Level I certification**—cumulative 3 months of training, minimum 75 TTE exams performed + 150 exams interpreted
	US cardiology trainees interpreting echocardiograms independently	**Level II certification**—cumulative 6 months of training, minimum 150 TTE exams performed + 300 exams interpreted
	US cardiologists completing a 3-year fellowship in echocardiography	**Level III certification**—cumulative 9 months of training, minimum 300 TTE exams performed + 750 exams interpreted
National Board of Echocardiography [26]	US and Canadian critical care physicians	**Critical care echocardiography (CCE) certification**—completion of a critical care fellowship, 20 + h of continuing medical education in echocardiography, minimum 150 TTE exams performed and interpreted
British Society of Echocardiography [27]	UK physicians performing bedside ultrasound	**Level 1 accreditation**—75 TTE exams performed over a 12-month period collected in a logbook encompassing a certain pathology, examination
	UK clinical echocardiographers	**Level 2 accreditation**—250 TTE exams performed over an 18-month period collected in a logbook encompassing a specific mix of cases, written assessment in theory and reporting, practical assessment, submission of 5 TTE video studies with report
British Society of Echocardiography, Intensive Care Society [28]	UK critical care physicians	**Adult critical care echocardiography accreditation (ACCE)**—250 TTE exams, written assessment, practical assessment
European Society of Intensive Care Medicine [29]	European critical care physicians	**European Diploma in advanced critical care EchoCardiography (EDEC)**—at least 3 courses, 100 TTE cases, and 35 TEE cases over a 2-year period, written examination, practical examination
European Society of Cardiology [30]	European sonographers and physicians	**European Association of Cardiovascular Imaging (EACVI) adult TTE certification**—completion of a written examination, minimum 250 TTE exams performed, submission of 6 TTE cases

## Methods

This systematic review conformed to the Preferred Reporting Items for Systematic Reviews and Meta-Analyses (PRISMA) guidelines [33].

### Data search

Our search strategy utilized PubMed, Embase, and Cochrane Library databases from inception until June 2020. The following search terms were used: “echocardiography” or “transthoracic echocardiography” or “TTE” or “bedside ultrasound” or “cardiac ultrasound”, and “doctors” or “physicians” or “residents” or “fellows” or “medical students” or “attending” or “intensivist” or “internist” or “hospitalist”, and “competence” or “competency” or “certification” or “accreditation” or “evaluation” or “assessment” or “curriculum”. These terms were identified in the title or abstract (PubMed and Embase) or in the title, abstract, or keywords (Cochrane). We also examined the lists of references from relevant studies and review articles for any additional articles that might have been missed in our initial search.

### Inclusion and exclusion criteria

Studies were included only if standardized training on FoCUS was provided followed by a formal assessment of competence, such as by expert review or comparison to an expert-performed echocardiogram. An expert was designated as a physician or sonographer with extensive training and/or certification in adult echocardiography. Included studies were required to have at least 5 learners who were medical students, trainees, or attending physicians without expertise or formal certification in transthoracic or transesophageal echocardiography. For inclusion, each study was required to outline the type and duration of training, describe which parameters were assessed, and identify a comparator for assessment of competency. Studies within pediatric populations and on non-physician learners were excluded.

### Study selection and data extraction

Titles and abstracts were assessed independently by two reviewers (LEG and PJL) and were included in the full text review if selected by either. The same two reviewers performed full text review, with discrepancies resolved by a third reviewer (MGC). Two authors (LEG and GAW) independently extracted the following data using a standardized form: number and training level of learners, ratio of learners to instructors during training, type and duration of training, total study duration, views and pathology taught, ultrasound device used, clinical setting, selection of subjects for assessment, parameters assessed, measurement of competency, and outcomes.

### Risk of bias assessment

The ROBINS-I tool [34] was used to assess risk of bias in our cohort of non-randomized studies of interventions. Risk of bias in seven pre-specified categories was independently assessed by two reviewers (LEG and GAW), with disputes resolved through joint discussion with a third reviewer (MGC).

### Study outcome

The primary outcome was the performance of medical students or physicians in acquiring and/or interpreting cardiac and hemodynamic parameters using FoCUS relative to that of expert echocardiographers.

### Data analysis

Summary tables are provided for included studies, accompanied by a qualitative discussion and evaluation of risk of bias. The relationship between three training parameters (didactic hours, hands-on practice hours, and scans performed) and reported level of agreement (kappa coefficient, *κ*) between learners and expert echocardiographers on identifying cardiac pathology was assessed by linear regression (SPSS version 24, IBM Corp.). Analysis was performed for parameters in which assessments were relatively uniform across studies, and for data sets containing ≥ 8 studies in order to minimize the likelihood of sampling error. The Pearson correlation coefficient (*r*) and *p* value for the linear fit were reported. The kappa coefficient (*κ*) was interpreted as [35]: perfect agreement (*κ* = 1), near perfect agreement (*κ* = 0.81 to 1), substantial agreement (*κ* = 0.61 to 0.8), moderate agreement (*κ* = 0.41 to 0.6), fair agreement (*κ* = 0.21 to 0.4), and slight to no agreement (*κ* = 0 to 0.2).

## Results

### Search results and study selection

**Fig. 1:**
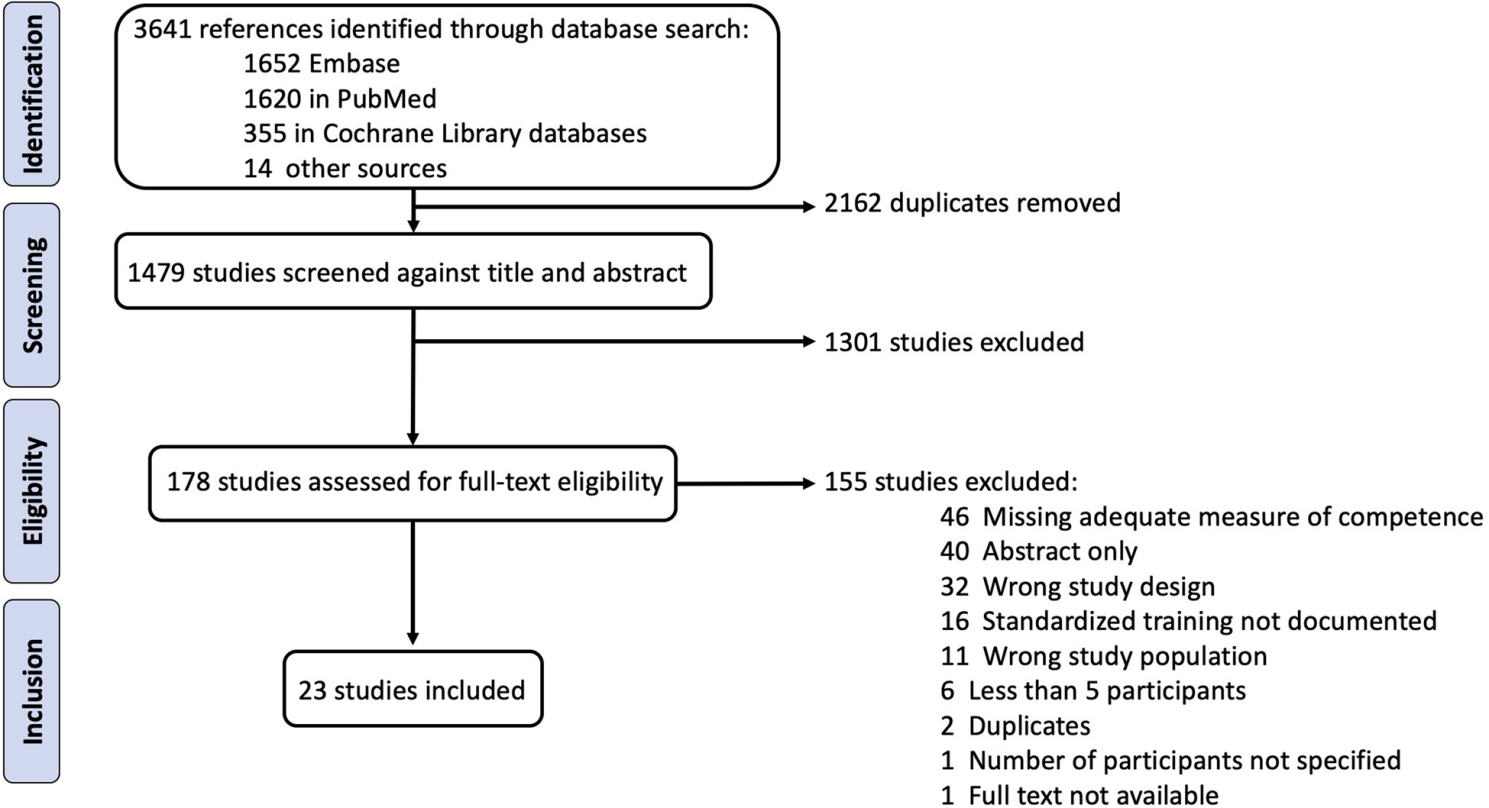
Flow diagram showing the selection of studies for inclusion

Our search yielded 1479 unique studies to be screened, of which 1301 were excluded, leaving 178 full-text studies to be screened. Of these, 23 met inclusion criteria and were included in this systematic review (Fig. 1). Many studies met multiple criteria for exclusion.

### Quality of included studies

All studies included in the analysis were non-randomized, observational studies of an intervention and thus were expected to have a substantial and unavoidable bias due to confounding. We identified several consistent sources of bias in the selection of participants, amount of training received by learners within each training program, number of scans performed, pre-existing knowledge of the clinical status of patient subjects, and interobserver variability. Bias was assessed for each study using the ROBINS-I tool (Table 2) [34].

**Table 2:** Risk of bias for each included study assessed using the ROBINS-I tool [26]

	Bias due to confounding	Bias due to selection of participants	Bias due to classification of interventions	Bias due to deviations from intended interventions	Bias due to missing outcome data	Bias due to outcome measurements	Bias due to selection of results reported	Overall risk of bias
Alexander 2004 [36]	Critical	Moderate	Low	Moderate	Serious	Moderate	Moderate	Critical
Beraud 2013 [37]	Critical	Serious	Moderate	Serious	Moderate	Serious	Moderate	Critical
Caronia 2013 [38]	Critical	Critical	Low	Moderate	Moderate	Serious	Moderate	Critical
Carrie 2015 [39]	Critical	Serious	Low	Moderate	Low	Serious	Moderate	Critical
Chisholm 2013 [40]	Critical	Critical	Low	Critical	Serious	Serious	Moderate	Critical
Croft 2006 [41]	Critical	Moderate	Low	Moderate	Low	Critical	Moderate	Critical
Farsi 2017 [42]	Critical	Critical	Moderate	NI	Moderate	Serious	Moderate	Critical
Ferrada 2011 [43]	Critical	Critical	Low	Low	Moderate	Critical	Moderate	Critical
Gaudet 2016 [44]	Critical	NI	Moderate	Moderate	Serious	Moderate	Moderate	Critical
Hellmann 2005 [45]	Critical	Moderate	Moderate	Moderate	Low	Moderate	Moderate	Critical
Johnson 2016 [46]	Critical	Moderate	Moderate	Moderate	Moderate	Serious	Moderate	Critical
Labbe 2016 [47]	Critical	Moderate	Low	Moderate	Moderate	Moderate	Moderate	Critical
Lucas 2009 [48]	Critical	Moderate	Low	Low	Low	Moderate	Moderate	Critical
Manasia 2005 [49]	Critical	NI	Serious	Serious	Low	Serious	Serious	Critical
Martin 2007 [50]	Critical	Serious	Moderate	Moderate	Low	Serious	Moderate	Critical
Mjolstad 2013 [51]	Critical	Moderate	Low	Serious	Low	Critical	Moderate	Critical
Mozzini 2014 [52]	Critical	Moderate	Serious	Critical	Serious	Critical	Serious	Critical
Ruddox 2013 [53]	Critical	Moderate	Low	Moderate	Moderate	Critical	Moderate	Critical
Ruddox 2017 [54]	Critical	Serious	Low	Moderate	Critical	Critical	Moderate	Critical
See 2014 [55]	Critical	Moderate	Low	Serious	Moderate	Moderate	Moderate	Critical
Smith 2018 [56]	Critical	Serious	Moderate	Critical	Moderate	Moderate	Moderate	Critical
Vignon 2011 [57]	Critical	Moderate	Low	Moderate	Moderate	Serious	Moderate	Critical
Yan 2018 [58]	Critical	Moderate	Low	Moderate	Low	Critical	Moderate	Critical

*NI* No information to make a fair assessment

### Study participants

Data were collected on a total of 292 learners across 23 studies (see Tables 3 and 4). Participants ranged from medical students to subspecialty physicians with up to 29 years of attending-level experience [43]. The most represented group was internal medicine residents (*n* = 174, 59.6%), followed by critical care fellows (*n* = 32, 11.0%), hospitalists (*n* = 27, 9.25%), emergency medicine residents (*n* = 23, 7.88%), emergency medicine attendings (*n* = 15, 5.14%), medical students (*n* = 10, 3.42%), intensivists (*n* = 6, 2.05%), trauma surgeons (*n* = 6, 2.05%), and anesthesia residents (*n* = 5, 1.71%). For the majority of learners, participation was on a voluntary basis. At least 9 learners (3.08%) across all included studies had some prior training in echocardiography, but none had expertise or formal certification.

**Table 3:** Characteristics of the learner population, training program, device used, and study duration for 23 included studies

	LEARNERS						ULTRASOUND DEVICE	STUDY DURATION
		Ratio of Learners to Instructors	Didactic hours	Hands-on hours	Total training hours	# of training scans		
*Alexander* 2004 [36]	20 IM residents	*Not specified*	1.5	1.5	3	*Not specified*	Phillips OptiGo	7 months
Beraud 2013 [37]	18 critical care fellows	18:1	8	15	23	25 exams (avg) Range: 18-32	Sonosite M-Turbo	1 year
*Caronia 2013* [38]	7 IM residents	1:1	5	3		15 exams (avg) Range: 5-31	Sonosite M-Turbo	1 month
*Carrie 2015* [39]	6 EM residents	*Not specified*	6	*Not specified*	6 + hands-on time^a^	*Not specified*	GE Vscan	3 months
Chisholm 2013 [40]	14 EM attendings	14:1	9.2	*Not specified*	9.2	8 exams (avg) Range: 3-13	Sonosite M-Turbo	5 months
Croft 2006 [41]	9 IM residents	*Not specified*	15	5	20	*Not specified*	Phillips OptiGo	1 month
*Farsi 2017* [42]	17 EM residents	*Not specified*	4	6	10	*Not specified*	Samsung SonoAce X8	3 months
Ferrada 2011 [43]	6 surgeons, 1 EM attending	7:1	1.16	0.5	1.66	1 exam	SonoSite S-ICU	*Not specified*
Gaudet 2016 [44]	6 critical care fellows	6:1	8	8	16	0 exams	Philips iE33 xMATRIX	*Not specified*
Hellmann 2005 [45]	30 IM residents	*Not specified*	0.75	*Not specified*	0.75 + self study^a^	3 exams	SonoSite 180	2 years
Johnson 2016 [46]	10 IM residents	*Not specified*	*Not specified*	*Not specified*	18	44 exams (avg)	SonoSite NanoMaxx or SonoSite EDGE	6 months
*Labbe 2016* [47]	5 IM residents	5:1	3.9	1.8	5.7	10 exams	Phillips HD11XE or Phillips CX50	5 months
	6 IM residents	6:1	5.6	2.7	8.3	27 exams		7 months
Lucas 2009 [48]	8 hospitalists	8:1	2	20	27	30 exams	MicroMaxx 3.4	3 months
Manasia 2005 [49]	6 intensivists	6:1	*Not specified*	*Not specified*	10	not specified	SonoHeart	9 months
Martin 2007 [50]	10 hospitalists	*Not specified*	6	*Not specified*	6 + self study^a^	5 exams	SonoSite Elite	4-7 months
Mjolstad 2013 [51]	6 IM residents	*Not specified*	4	*Not specified*	4	95 exams (median) Range: 80-225	GE Vscan 1.2	2.5 months
*Mozzini 2014* [52]	23 IM residents	*Not specified*	*Not specified*	*Not specified*	9	5 exams	Philips Envisor	9 months
	12 IM residents	*Not specified*	*Not specified*	*Not specified*	18	5 exams		
*Ruddox 2013* [53]	26 IM residents	5:1 or 6:1	1	1	2	*Not specified*	GE Vscan	9 months
*Ruddox 2017* [54]	20 IM residents	2:1, 3:1, or 4:1	1.5	2.5	4	*Not specified*	GE Vscan	6 months
See 2014 [55]	7 critical care fellows	Online learning	10	2.5	12.5	5 exams	Philips Sparq	1 year
Smith 2018 [56]	9 hospitalists	9:1	8+	*Not specified*	30.6 (avg)	*Not specified*	Philips Sparq	6 months
*Vignon 2010* [57]	5 anesthesia residents, 1 IM resident	3:1	6	6	12	10-12 exams	Philips OptiGo	3 months
Yan 2010 [58]	10 medical students	*Not specified*	7	3	10	*Not specified*	GE Vscan	1 months

Italicized studies reported kappa agreement values for LV systolic function and/or pericardial effusion and were therefore included in quantitative analysis

*IM* Internal medicine, *EM* Emergency medicine, *avg* Average

^a^Duration not specified

**Table 4:** Characteristics of ultrasound skill assessment and overall findings for 23 included studies

	SUBJECTS ASSESSED				FINDINGS	
		Views	Pathology Assessed	Comparator		
*Alexander 2004* [36]	533 patients (mixed: ICU, intermediate care unit, clinic)	PLAX, PSAX, A4 (+ color doppler)	LV function (EF), MR, aortic valve mobility, pericardial effusion	TTE performed by experienced sonographer and interpreted by echocardiographer, as assessed by experienced observer	**Acquisition:** learners obtained satisfactory images to assess LV function in 98.7% of patients, MR in 92.7%, aortic valvular disease in 89.7%, and pericardial effusions in 97.9%	**Interpretation**: learners showed fair agreement with TTE for identifying MR (κ = 0.31), aortic valvular disease (κ = 0.31) and moderate agreement for pericardial effusion (κ = 0.51) and LV dysfunction (κ = 0.51)
Beraud 2013 [37]	5 simulated cases	PLAX, PSAX, A4, SC4, IVC	LV function, RV dilation, RV function, pericardial effusion	Simulator performance by novice medical students and expert sonographers	**Acquisition:** novices took longer to scan (358 ± 170s) than fellows (136 ± 63s), and fellows took longer than experts (38 ± 6s)	**Interpretation**: one diagnosis (RV dysfunction) was missed by one fellow (5.5%) + 3 novices (33%); experts were significantly faster (18 ± 7s) than fellows (72 ± 38s), who were significantly faster than novices (185 ± 86s)
*Caronia 2013* [38]	102 patients (mixed: ICU, intermediate care unit)	PLAX, PSAX, A4, SC4 (+ color doppler)	LV function (EF, WMAs), RV strain or diastolic collapse, septal defects, valvular lesions, thrombi, aneurysm, pericardial effusion	Findings on TTE performed by sonographer and interpreted by cardiologist, as assessed by a trained fellow	**Interpretation**: learners correctly identified 95% of patients with systolic dysfunction (κ = 0.67), 85% of patients with pericardial effusion (κ = 0.60), but only 41% patients with RV strain (κ = 0.38); valvular pathologies were identified with moderate agreement (κ = 0.50 - 0.52) as were WMAs (κ = 0.49)	
*Carrie 2015* [39]	180 ED patients	PLAX, PSAX, A4, SC4, IVC, lung, abdomen	LV size (LVH) + function, RV dilation, IVC size, pericardial effusion, pleural effusion, consolidations, interstitial fluid, abdominal pathology	Focused exam performed by experienced physician board-certified in ultrasound or echocardiography	**Acquisition:** learners took longer to perform exams than experts, but exam time decreased from 14.5 min (0-10 exams performed) to 10 min (10-20 exams) to 8 min (20-30 exams), while experts required 4-6 min for exams	**Interpretation**: agreement with experts improved when comparing learner performance over the first 10 exams performed (exams 0-10) with the last 10 exams performed (exams 20-30) for assessment of LV function (κ=0.77 to κ=0.92), LVH (κ=0.67 to κ=0.9), IVC dilation (κ=0.6 to κ=0.88) and collapse (κ=0.3 to κ=0.76), while agreement peaked after completion of 10-20 exams for assessing RV dilation (κ=0.78 to κ=0.83) and pericardial effusion (κ=0.74 to κ=0.9)
Chisholm 2013 [40]	1 healthy volunteer	PLAX, PSAX, A4, SC4, IVC	none	Evaluation of images by cardiologist board-certified in echocardiography	**Acquisition**: 85% were able to achieve acceptable PLAX + PSAX views within 120s, 70% and 57% were able to achieve SC4 and A4 views (respectively), and <50% obtained IVC view; SC4 and A4 views were more likely to be obtained by those who completed > 45 practice studies	
Croft 2006 [41]	72 clinic patients	PLAX, PSAX, A4, A2 (+ color doppler)	LV size + function (WMAs, LVH), valvular lesions, pericardial effusion	Focused exam performed by a level 3 certified echocardiographer	**Acquisition:** learners obtained diagnostic images in 94% of patients; A2 view was most difficult and was imaged adequately in 68% of patients while PLAX, PSAX, and A4 were imaged adequately in 96%, 92%, and 94%, respectively	**Interpretation:** learners correctly identified 93% of major findings (PPV = 93%, NPV = 99%) and 78% of minor findings (PPV = 97%, NPV = 93%)
*Farsi 2017* [42]	205 ED patients	PLAX, PSAX, A4, SC4	LV function (EF by EPSS or Quinones equation, WMAs), RV dilation, pericardial effusion	Findings on TTE performed by a cardiologist	**Interpretation**: all major pathologies were identified with >90% accuracy and with near perfect agreement to cardiologists, including low LVEF (κ=0.85), WMAs (κ=0.83), RV dilation (κ=0.86) and pressure overload (κ=1.00), + pericardial effusion (κ=0.83)	
Ferrada 2011 [43]	51 ICU patients	PLAX, PSAX, A4, SC4, IVC	LV function, IVC size, pericardial effusion	TTE performed and interpreted by a cardiologist	**Acquisition:** the A4 was the only view that could not be obtained in all patients (84.3%)	**Interpretation**: there was 100% correlation between learners and cardiologists on global heart function and contractility
Gaudet 2016 [44]	36 ICU patients	PLAX, PSAX, A4, SC4, IVC (+ M-mode)	none	Performance on 1st and 2nd exams, 10th and 11th exams, and 19th and 20th exams as assessed by a level 3 certified intensivist	**Efficiency**: efficiency improved incrementally after the first 10 studies (1.55 to 2.48) and by a greater extent than after 10 to 20 studies (2.48 to 2.61); efficiency was lower for trainees compared to experts at all intervals	**Workload**: mental and physical demand, time, effort, frustration, and anxiety decreased throughout all assessment intervals, with the greatest reduction after completing the first 10 studies
Hellmann 2005 [45]	229 floor patients	PLAX, PSAX, A4, A2 (+ color doppler)	LV size (thickness) + function, septal thickness, LA size, valvular lesions, pericardial effusion, aortic size	Review of TTE and focused exam by an experienced, level 3 certified cardiologist	**Acquisition**: image acquisition improved over time as more scans were completed (up to 22 scans); the A2 view had the slowest rate of learning while the PSAX was learned at the fastest rate	**Interpretation**: interpretation accuracy improved over time when performing up to 22 scans; measurement of LV diastolic size and identification of pericardial effusions were learned fastest while identifying AS, MR, and measuring septal wall thickness had the slowest rate of learning
Johnson 2016 [46]	178 patients (mixed: ICU, intermediate care unit, floor)	PLAX, PSAX, A4 or A5, A2, ALAX, SC4	LV function	TTE performed by trained sonographer and interpreted by a level 2 or 3 certified cardiologist	**Acquisition:** 100% of learner-performed exams were adequate to characterize LV systolic function	**Interpretation:** learners identified impaired LV function with substantial agreement (κ=0.77, sens=0.91, spec=0.88) with experts, similar to the interobserver variability among echocardiographers within the study institution (κ=0.78); learners had the lowest sens and spec for identifying mild/moderate LV dysfunction (sens=0.70, spec=0.86)
*Labbe 2016* [47]	115 ICU patients	PLAX, PSAX, A4, SC4, IVC (+ color, pw, cw doppler)	LV function (EF, WMAs, LV outflow tract velocity time integral, filling pressure, E/A + E/e' ratio), AV, MV, RV dilation, IVC size, pericardial effusion	Focused exam performed by a level 3 certified cardiologist, as assessed by two independent cardiologists	**Acquisition**: learners in the shorter training group obtained at least one optimal view in 83% of exams, compared to 91% by those who received additional training, while experts obtained at least one optimal view in 98% and 94% of exams; no difference in exam duration between the 2 learner groups (22±8 min, 22±10 min, respectively), but both groups took longer than experts to perform exams (12±6 min, 13±7 min)	**Interpretation**: learners in the shorter training group had more unanswered questions than experts (13% vs 7%), while those with additional training had a similar # of unanswered questions as experts (9% vs 8.5%); those who received additional training interpreted RV dilation, valvular pathology, and calculated aortic peak velocity, E/A ratio, and E/e' ratio with greater accuracy than those who received shorter training
	108 ICU patients		Above plus septal deviation			
Lucas 2009 [48]	314 patients (mixed: ICU, floor, short stay unit)	PLAX, PSAX, A4, A2, IVC	LV size + function (LVH), MR, LA size, IVC size, pericardial effusion	TTE performed by experienced sonographers and interpreted by a level 2 echocardiograpy certified cardiologist	**Acquisition**: leaners were able to obtain adequate imaging on 94-98% of intended assessments	**Interpretation**: learners were best at identifying severe MR (sens = 100%, spec = 83%), pericardial effusions (sens = 100%, spec = 95%), LA dilation (sens = 90%, spec = 74%), LV dysfunction (sens = 85%, spec = 88%), and less skilled at identifying LVH (sens = 70%, spec = 73%) and dilated IVC (sens = 56%, spec = 86%)
Manasia 2005 [49]	90 ICU patients	PLAX, PSAX, A2, A4	LV function (WMAs), pericardial effusion	Focused exam performed by cardiologist, and assessment of learners’ images by a cardiologist	**Acquisition**: learners were able to successfully perform a TTE exam in 94% of patients	**Interpretation**: 84% of exams were correctly interpreted by learners
Martin 2007 [50]	354 floor patients	PLAX, PSAX, A4, SC4 (+ color, pw doppler)	LV size (wall thickness) + function (E/A ratio), LA size, valvular lesions, vegetations, pericardial effusion, aortic size	Image quality on focused TTE performed by echocardiography technician, assessed by an independent cardiologist and compared against TTE; interpretation of pre-recorded focused exams by cardiology fellows	**Acquisition**: learners obtained less optimal images than technicians across all views (80.6% agreement with TTE image quality vs 98.9% agreement), particularly on the A4 view (difference of 33.6%)	**Interpretation**: learner measurements were less accurate than technicians (80.2% vs 89.9% agreement); learners were best at assessing aortic root size + diastolic LV size (differences of 5.6% and 6.5%, respectively) and least accurate for systolic LV size (13.1% difference); cardiology fellows were no better than learners at interpreting E/A ratio, MR, and AS but were more skilled at interpreting LV function, AR, pericardial effusions, and vegetations
Mjolstad 2013 [51]	199 floor patients	PLAX, PSAX, A4, A2, ALAX, IVC, lung (+ color doppler)	LV function (WMAs), RV function (systolic excursion), RV dilation, septal flattening, valvular lesions, LA size, IVC size, pericardial effusion, aortic size	TTE performed by cardiologist or to findings on computed tomography imaging for detection of pleural effusion and aortic size	**Acquisition**: learners were able to assess LV function, pericardial effusion, + pleural effusions in >95% of patients, were able to assess RV function, LA size, MV, + AV in > 85% of patients, TV + IVC in > 75% of patients, and abdominal aorta + PV in only 50% and 49% of patients, respectively	**Interpretation**: learners interpreted LV function, pleural effusions, and pericardial effusions with very strong correlation with experts (r ≥ 0.83); AS, AR, + aortic aneurysms with strong correlation (r ≥ 0.67); WMAs, LA dilation, IVC size, TR, and MR with moderate correlation (r ≥ 0.53)
*Mozzini 2014* [52]	15 floor patients	PLAX, PSAX, A4, SC4, IVC, supra-sternal	LV size + function, RV dilation, IVC size, pericardial effusion	TTE performed by sonographer and interpreted by either a cardiologist or a hospital certified in echocardiography	**Acquisition**: students who received 18 hours of training were more skilled at obtaining parasternal and apical views compared to those who received only 9 hours; learners required longer (7±1 min) to perform each exam during the first 3 days compared to the second 3 days (4±0.5 min)	**Interpretation**: students who received 18 hrs of training were more skilled at interpretation compared to those who received 9 hrs; after 18 hrs learners showed substantial agreement with experts on identifying pericardial effusions (κ=0.71), global LV function (κ=0.2 to 0.77), + atrial size (κ=0.66), moderate agreement for LV (κ=0.54) + RV (κ=0.56) enlargement and valvular pathology (κ=0.56), and fair agreement for IVC size (κ=0.35), WMAs (κ=0.35), + aortic size (κ=0.28)
	30 floor patients	Above + M-mode, color doppler	LV size + function (EF by Simpson's method, WMAs, MAPSE), RV size + function, valvular lesions, IVC size, pericardial effusion, aortic size			
*Ruddox 2013* [53]	303 ICU or ED patients	A4, A2, ALAX	LV size + function (EF, WMAs), RV function, LA size, valvular lesions, aortic dilation, IVC size, pericardial effusion	Findings on focused exam performed by a level 3 certified echocardiographer	**Interpretation**: learners identified LVEF < 40% (κ = 0.53), LV dilation (κ = 0.43), WMAs (κ = 0.64), pericardial effusion (κ = 0.4), valvular abnormalities (avg κ = 0.43), + dilated IVC (κ = 0.17); overall accuracy improved over time from 0-9 exams performed (κ = 0.22) to 20-29 exams performed (κ = 0.38), and only marginally after 30-35 exams (κ= 0.41)	
*Ruddox 2017* [54]	60 floor patients	PLAX, A4 (+ color doppler)	LV size + function (EF, WMAs), MR, AR, aortic dilation, pericardial effusion	Findings on focused exam performed by a level 3 certified echocardiographer	**Interpretation:** learners identified LVEF < 40% (κ = 0.7), LV dilation (κ = 0.75), and LA dilation (κ = 0.66) with substantial agreement with TTE, RV pathology (κ = 0.42-0.48), MR (κ = 0.56), and WMAs (κ = 0.44) with moderate agreement, and pericardial effusion (κ = 0.30) + AR (κ = 0.35) with fair agreement	
See 2014 [55]	318 ICU patients	PLAX, PSAX, A4, SC4, IVC (+ M-mode, color doppler)	LV function (EF by Simpson's method), RV dilation, MR, IVC size, pericardial effusion	Review of images by an intensivist experienced in critical care ultrasound	**Acquisition:** views obtained improved from 40% acceptable after 1-10 exams to 91% acceptable after at least 30 exams; learners took 21.3 ± 9.5 min for each exam and 18.9 ± 7 min for each after the first 30 studies; scanning duration decreased by 0.14 min (CI 0.10 - 0.18 min) after each successive study	**Interpretation**: pathologies were accurately assessed in > 80% after 11-20 exams, and > 90% after 30 exams for all pathologies except for estimation of LVEF fraction (85% after 30 exams)
Smith 2018 [56]	3 standardized patients	PLAX, PSAX, A4, SC4, IVC	none	Focused exam performed by a level 1 certified cardiology fellows and by experienced sonographers, interpreted by cardiologists (2 independent evaluators)	**Acquisition**: learners obtained comparable quality images as fellows for PLAX and SC4 views, but fellows obtained higher quality images for PSAX, A4, and IVC views; learners and fellows exam durations were similar (15.3 min and 13.8 min, respectively)	**Efficiency**: there was no difference in efficiency between learners and senior cardiology fellows, but experts consistently performed better than cardiology fellows and learners
*Vignon 2010* [57]	201 ICU patients	PLAX, PSAX, A4, SC4, IVC	LV size + function (EF, WMAs), RV size + function, IVC size, pericardial effusion	Focused exam performed by an experienced, level 3 certified intensivist	**Acquisition**: leaners performed longer exams than experts (7±2.5 min vs 3±1 min) and obtained fewer views (82% vs 88%); image quality was significantly better when performed by experts for the PSAX view and similar (good to excellent) for all others	**Interpretation**: learners had perfect agreement with experts in identifying tamponade (κ=1), near-perfect agreement when interpreting LV function (κ=0.84) and LV dilation (κ=0.90), and substantial agreement for identifying RV dilation (κ=0.76), IVC dilation (κ=0.79) and collapse (κ=0.66), + pericardial effusion (κ=0.79)
Yan 2010 [58]	107 patients (mixed: floor, clinic)	PLAX, PSAX, A4, SC4 (+ color doppler)	Valvular lesions	Findings on focused exam performed by trained nurse or cardiologist	**Interpretation**: learners had moderate agreement (κ=0.45) with experts when identifying valvular findings and were most skilled at identifying MS, MR, and AS, and substantially less skilled at identifying AR (κ=0.23)	

Certification levels of experts used for comparison were noted if specified in the text and refer to the ACC/ASE certification levels (see Table 1). Italicized studies reported kappa agreement values for LV systolic function and/or pericardial effusion and were therefore included in quantitative analysis.

*PLAX* Parasternal long axis, *PSAX* Parasternal short axis, *A4* Apical 4-chamber, *SC4* Subcostal 4-chamber, *IVC* Inferior vena cava, *A2* Apical 2-chambere, *ALAX* Apical long axis (apical 3-chamber), *pw* Pulsed wave, *cw* Continuous wave, *LV* Left ventricle, *EF* Ejection fraction, *MR* Mitral regurgitation, *RV* Right ventricle, *WMAs* Regional wall motion abnormalities, *LVH* Left ventricular hypertrophy, *EPSS* E-point septal separation, *AV* Aortic valve, *MV* Mitral valve, *LA* Left atrium, *AR* Aortic regurgitation, *MAPSE* Mitral annular plane systolic excursion, *RVSP* Right ventricular systolic pressure, *ICU* Intensive care unit, *ED* Emergency department, *LVEF* Left ventricular ejection fraction, *HTN* Hypertension, *sens* Sensitivity, *spec* Specificity, *κ* Cohen’s kappa coefficient

### Training format and duration

All studies had a standardized training program that included some combination of didactic and practical hands-on learning. Where reported, the didactic component ranged from 45 min to 18 h, and from 7 to 80% of the dedicated training time. Didactics included a component of in-person lectures, review of pre-recorded cases, and/or bedside demonstration in 21 of 23 studies (91%) and consisted of remote learning only with handouts or online modules in 2 of 23 studies (8.7%). Practical learning was reported either as a duration of time spent in small groups or 1-on-1 performing supervised echocardiograms, or as the number of supervised exams or exams performed independently with feedback. Where reported, the time spent on practical training ranged from 30 min to 20 h or from between 1 and 50 exams, with the exception of one study in which learners were encouraged to perform 100 independent exams as part of their training [51].

### Subjects for assessment

Learners performed FoCUS on a total of 3794 subjects, which included 3785 patients, 4 healthy volunteers, and 5 simulated patient cases. Patients were examined in a variety of clinical settings, including the intensive care unit (*n* = 1077, 28.5%), inpatient medicine floor (*n* = 1002, 26.5%), intermediate care unit (*n* = 408, 10.8%), emergency department (*n* = 385, 10.2%), outpatient clinic (*n* = 257, 6.79%), and short-stay unit (*n* = 175, 4.62%). A total of 524 patients (13.8%) were on mechanical ventilation at the time of the exam. Clinical setting was not specified for 481 patients (12.7%). Most patients were selected for study inclusion based on having a clinical indication for FoCUS, and many were excluded due to the presence of injuries requiring immediate intervention, inability to tolerate repositioning, the sonographers’ inability to obtain adequate windows, or a prolonged duration (typically > 48 h) between learner and expert examinations.

### Parameters for assessing competency

Learners were assessed on their skills in both acquiring and/or interpreting images. Parameters of acquisition ability included whether or not learners were able to obtain adequate images to make a diagnosis, the time required to obtain images, a subjective assessment of image quality, or an efficiency score (quality/time). One study also reported self-perceived workload for performing FoCUS [44]. Parameters of interpretation ability included accuracy in quantitative measurements (chamber or vessel sizes, ejection fraction, E/A ratio) and diagnostic accuracy (normal or abnormal function, presence or absence of pathology). Competency in these areas was assessed by comparison against the performance of an expert echocardiographer. This was typically a board certified cardiologist or a physician who had completed level 2 or 3 certification by the American Society of Echocardiography, although in two studies this was a cardiology fellow [50] or intensivist [55] with formal training and experience in echocardiography but without certification. Ideally, exams performed by learners were compared to a similar exam performed by an expert, with both exams performed using either a portable or traditional ultrasound. However, only in 8 of the 23 studies [40, 41, 45, 47, 49, 50, 56, 57] were the learner’s exam compared to another focused exam performed on the same or very similar type of device. Most studies included comparison of a learner-performed FoCUS exam with a standard TTE, and often with the learner performing the exam with a portable device with limited functionality and poorer image resolution than a traditional ultrasound machine. One study [37] compared learner and expert performance on an ultrasound simulator, while two others examined healthy volunteers [40, 56].

### Quantitative assessment of training parameters

**Fig. 2:**
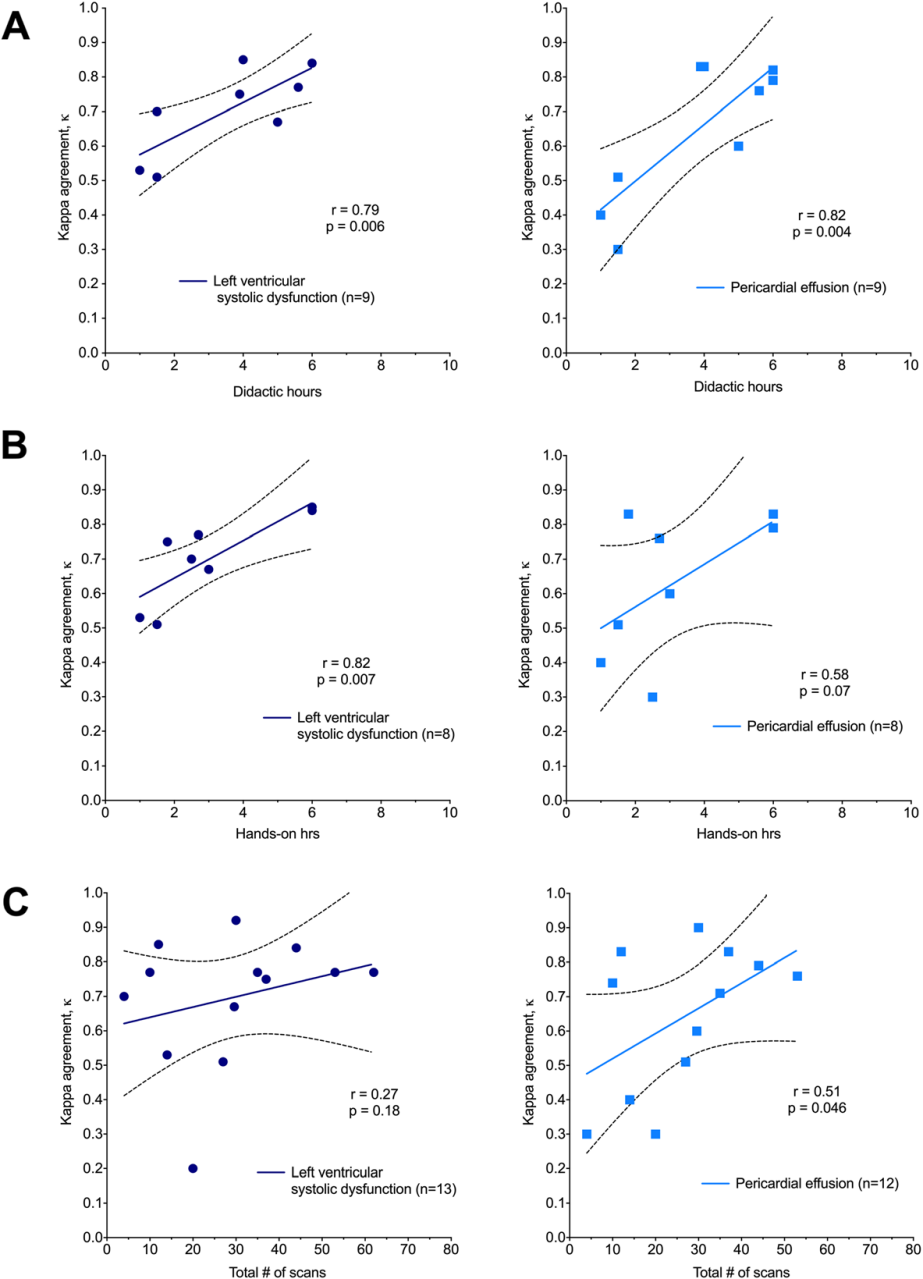
Relationship between **a** number of didactic hours, **b** number of hands-on practice hours, and **c** number of scans performed during a standardized training phase on learner agreement with expert echocardiographers for the detection of left ventricular systolic dysfunction (left panel, navy) and pericardial effusions (right panel, light blue). The Pearson correlation coefficient (*r*) and *p* value for the linear fit are reported for each data set, and regression lines are shown with 95% confidence intervals (dashed lines). Agreement is expressed by the kappa coefficient, κ

Of the 23 studies included in this review, 11 calculated a kappa coefficient (κ) for inter-rater reliability between learner and expert interpretation of at least one cardiac ultrasound finding and could be included for quantitative analysis. The most frequently assessed pathologies were left ventricular (LV) systolic dysfunction and pericardial effusion, followed by regional wall motion abnormalities, valvular abnormalities, and hypovolemia. LV systolic function and the presence of pericardial effusion were assessed in at least 8 studies, providing the largest sample sizes for meta-analysis. The other parameters had limited sample sizes with measures that were relatively less uniform across studies. The level of agreement with experts on learner assessment of LV systolic function (Fig. 2, left panel) and pericardial effusion (Fig. 2, right panel) is shown based on the number of didactic hours (Fig. 2a), number of hands-on practice hours (Fig. 2b), and total number of exams performed (Fig. 2c). Learners achieved near perfect agreement (*κ* > 0.8) with expert echocardiographers on the assessment of LV systolic function after 6 didactic hours and 6 h of hands-on training, and substantial agreement (*κ* > 0.6) after 2 h of didactics and 2 h of hands-on training. There was no correlation between number of scans performed and agreement with experts on the identification of LV systolic dysfunction. Learners achieved substantial agreement (*κ* > 0.6) with experts on the identification of pericardial effusion after 3 h of didactics, 3 h of hands-on training, and at least 25 scans. For the assessment of LV systolic function, agreement between learners and experts correlated with the amount of time (1 to 6 h) spent on didactics (*r* = 0.79, *p* < 0.05) and performing hands-on practice (*r* = 0.82, *p* < 0.05). For the identification of pericardial effusion, agreement between learners and experts correlated with the amount of time (1 to 6 h) spent on didactics (*r* = 0.82, *p* < 0.005) and the number of scans performed in each study (*r* = 0.51, *p* < 0.05).

## Discussion

FoCUS is intended to provide qualitative or semi-quantitative assessment of major cardiac abnormalities, such as identifying LV systolic dysfunction, pericardial effusion, or valvular abnormalities [59]. As a goal-directed tool, data obtained needs to be reliable as it is used to guide immediate clinical management. Thus, the development of a FoCUS training platform that ensures competency is necessary for safe and meaningful use. Our systematic review has shown that existing training programs vary substantially in their duration of training (45 min to over 20 h), type of training provided, skills taught, and clinical setting in which FoCUS skills were assessed. Our analysis also showed that a short duration of training, i.e., 2–3 h didactics and 2–3 h of hands-on training, may be sufficient for most learners to achieve substantial agreement with experts in identifying two major cardiac abnormalities: LV systolic dysfunction and pericardial effusion. Meanwhile, near perfect agreement (*κ* > 0.8) for detecting these abnormalities could be achieved after 6 h of didactics and 6 h of hands-on training. Identification of other pathologies, particularly wall motion abnormalities, valvular lesions, and IVC enlargement, was often more difficult, and most learners were only able to achieve fair to moderate agreement with experts after brief training.

Many studies included in our review involved comparison of data obtained through FoCUS exams performed using a small portable or handheld device to data obtained from a TTE performed using ultrasound machines with high resolution and advanced features. FoCUS is not performed for the same diagnostic purpose, nor should it be expected to match the precision of a comprehensive TTE. Yet we felt that comparison to a well-established standard was likely to be the most reliable metric to assess learner competency and that results yielded from this higher benchmark should be interpreted within a margin of non-inferiority. FoCUS training should also include education on the intended use and inherent limitations of FoCUS versus TTE.

Our review examined the effect of three training parameters on learner performance. We showed that substantial agreement (*κ* > 0.6) between learners and experts on the assessment of LV systolic function could be achieved with only 2 h each of didactic and hands-on practice and a minimum (4–10) number of scans. Similarly, substantial agreement with experts on the identification of pericardial effusion could be achieved with only 3 h each of didactic and hands-on practice. The greater amount of time required for identifying pericardial effusions may be due to misidentification of pericardial fat as an effusion, or to the fact that small effusions can be missed in some views. Regardless, these findings are impressive, given that only moderate (*κ* > 0.4) to substantial (*κ* > 0.6) agreement exists between trained experts for assessments of LV function by FoCUS [60]. We also show that learner performance for identifying LV systolic dysfunction improves with time spent on didactics and time spent performing hands-on practice, at least for up to 6 h each, whereas the total number of scans performed did not correlate with improvement in identifying LV dysfunction. This may be due to the fact that there was already substantial agreement (*κ* > 0.6) between learners and experts after very few (4–10) scans. Also, identifying LV dysfunction by FoCUS is a skill that may be best taught through a combination of didactics and supervised practice, while the actual number of exams performed may be less important. In contrast, identification of pericardial effusion improved with time spent on didactics as well as with the number of scans performed, and substantial agreement with experts could be achieved after 25 scans. This suggests that the detection of pericardial effusion is a skill that is gained through additional experience rather than supervised practice and supports the completion of between 20 and 30 focused exams for achieving competency in FoCUS as recommended by existing governing bodies (Table 1). Overall, our quantitative findings confirm that learners may be able to achieve reasonable competency using ultrasound to assess LV function and identify pericardial effusion after a very short (4–6 h) duration of training that includes equal portions (2–3 h each) of didactic and hands-on learning. Our findings also suggest that a small number of scans (20–30) may be sufficient for learners to gain basic competency in FoCUS.

To our knowledge, ours is the first systematic review and meta-analysis to be published on training in FoCUS. A prior systematic review by Rajamani et al. [61] examined 42 studies with an aim of evaluating the quality of point-of-care ultrasound training programs and their ability to determine competence. Roughly half of all studies did not include a comparator group against which to assess learner competency. Another prior systematic review by Kanji et al. [32] examined 15 studies in the critical care setting, most of which assessed learning based on pre- and post-training test scores and also did not include assessment of competency against an accepted standard as was required in our review. In addition to requiring a comparator for assessing competency, we also took a broader approach in examining the training of a diverse group of learners. As FoCUS adoption continues to expand, we wanted to report findings that might guide appropriate guidelines for the education of providers from different backgrounds and skill levels.

Our review is the first to provide quantitative evaluation of the impact of various training parameters on learner performance. While established curricula exist for FoCUS training in critical care and in emergency medicine, such standards do not currently exist for other specialties. By including a heterogenous population of learners in our review, we hope that the findings may be generalizable to learners in other specialties such as internal medicine, anesthesiology, and general surgery who may be examining patients in settings ranging from outpatient clinic, in the operating room, or post-operatively in the hospital wards. Studies also ranged in their scope of training and parameters assessed, emphasizing that the determination of competency in performing and interpreting FoCUS is a challenging distinction that depends heavily on the clinical context. Because the goal of FoCUS will vary based on the clinical context to which they are applied, the specific metrics for competency will also vary [17]. For example, sensitivity for the detection of a reduction in left ventricular ejection fraction needs to be high in outpatient settings, such as in the study by Croft et al. [41], when determining the need for specialty referral and tailored management of chronic conditions. Meanwhile, a lower sensitivity is likely acceptable in the emergency department, such as in the studies by Farsi et al. [42] and Carrie et al. [39], when determining the presence of a cardiogenic cause for hemodynamic instability.

When considering the wide range of potential clinical applications for FoCUS, it is important to recognize that training clinicians with different skill levels for the use of FoCUS in a variety of settings is unlikely to be successful with a single standardized curriculum. Rather than content-based training that uses completion of a set of material as an endpoint, a competency-based program recognizes that learners will progress at different speeds and that some will require additional material to reach the same level of competency. Competency-based programs enable learners to move through topics at their own pace, progressing when they are comfortable with a new skill and deemed competent by their supervisor(s). This form of training has been successful for teaching other clinical skills such as central line placement and orotracheal intubation, in which clinical competency is not strictly linked to a number of lines placed or intubations performed and no formal accreditation is needed. The future practice of FoCUS may benefit from a convergence on competency-based training that is tailored to a particular application and/or specialty, rather than from pursuit of formal accreditation across specialties.

When considering the most effective ways to train physicians on the use of FoCUS, it is also important to recognize that the co-existent clinical demands on physician-learners can impede skill acquisition. Some of the strategies to support learners that were adopted by the studies in this review include offering one-on-one or small group sessions for additional supervised practice, providing supervision during clinical application, and establishing processes that give learners access to ongoing feedback from experts. Flexibility in training availability and integration of FoCUS practice with existing clinical workflows were two recurring strategies that seemed to cater to the needs of physician-learners.

The need to train new generations of physicians in adult FoCUS presents the opportunity for future study in this field. An important consideration when designing a training program is the prevention of skill decay, which has been noted to occur rapidly (within 1–3 months) after the completion of a brief training program [62]. One study [56] found that learners retained their imaging skills at 6 months post-training, but there was no data on skill retention beyond 6 months in any included studies. The duration of the training phase may be inversely related to the rate of decay, suggesting that longitudinal support through deliberate practice and mentored review may help learners to retain their skills [56]. By making ultrasound devices readily available and easily accessible within clinical environments, physicians can develop ways to incorporate FoCUS into their daily practice. Training programs must find ways to support learners beyond the initial training period in a manner that is structured yet flexible.

## Limitations

It is important for the reader to recognize that all of the studies identified were non-randomized, observational studies with critical levels of bias. First, selection bias was often evident in both the selection of participants, many of whom were volunteers, and the selection of patient subjects for exams. For example, patients requiring urgent evaluation and treatment are those who are also most likely to benefit from rapid, point-of-care ultrasound, and yet many of these patients were excluded from learner examinations. Three studies reduced subject selection bias by using standardized patients or an ultrasound simulator [37, 40, 56], but at the expense of external validity. Second, few studies [39, 44, 45, 50, 55] acknowledged exams performed by each learner as dependent data points, and even fewer accounted for this through the use of linear modeling [45, 50]. Third, most studies were conducted in actual clinical settings, where time constraints, patient factors, and learner motivation are expected to introduce bias into the results. And lastly, while we report the minimum hours required for learners to detect LV systolic function and identify the presence of pericardial effusion, we were unable to determine the minimum training period required to achieve competency in other aspects of cardiac assessment due to insufficient data.

## Conclusion

FoCUS is an important diagnostic tool and will likely soon be considered a standard skillset for any practicing physician. A formal training program that includes 2–3 h of didactic learning, 2–3 h of hands-on training, and requiring 20–30 scans is likely to be adequate for most learners to achieve competency in the detection of gross LV systolic dysfunction and pericardial effusion. Additional training is necessary for skill retention, efficiency in image acquisition, and the detection of more subtle abnormalities. The finding that reasonable proficiency can be obtained after only brief formal training should encourage physicians at any career level to pursue training in FoCUS.

## Data Availability

Aggregated data available by request

## Abbreviations

FoCUS: Focused cardiac ultrasound
*κ*: Kappa coefficient
LV: Left ventricular
NI: No information
*r*: Pearson correlation coefficient
PRISMA: Preferred Reporting Items for Systematic Reviews and Meta-Analyses
TTE: Transthoracic echocardiography

## References

[1] Clement GT (2004). Perspectives in clinical uses of high-intensity focused ultrasound. Ultrasonics..

[2] Nelson BP, Sanghvi A (2013). Point-of-care cardiac ultrasound: feasibility of performance by noncardiologists. Glob Heart.

[3] Cowie B, Kluger R (2011). Evaluation of systolic murmurs using transthoracic echocardiography by anaesthetic trainees. Anaesthesia..

[4] Frederiksen CA, Juhl-Olsen P, Andersen NH, Sloth E (2013). Assessment of cardiac pathology by point-of-care ultrasonography performed by a novice examiner is comparable to the gold standard. Scand J Trauma Resusc Emerg Med.

[5] Spencer KT, Kimura BJ, Korcarz CE, Pellikka PA, Rahko PS, Siegel RJ (2013). Focused cardiac ultrasound: recommendations from the American Society of Echocardiography. J Am Soc Echocardiogr.

[6] Canty DJ, Royse CF, Kilpatrick D, Bowman L, Royse AG (2012). The impact of focused transthoracic echocardiography in the pre-operative clinic: transthoracic echocardiography in the pre-operative clinic. Anaesthesia..

[7] Kratz T, Steinfeldt T, Exner M, Dell´Orto MC, Timmesfeld N, Kratz C (2017). Impact of focused intraoperative transthoracic echocardiography by anesthesiologists on management in hemodynamically unstable high-risk noncardiac surgery patients. J Cardiothorac Vasc Anesth.

[8] Orme RML, Oram MP, McKinstry CE (2009). Impact of echocardiography on patient management in the intensive care unit: an audit of district general hospital practice. Br J Anaesth.

[9] Hall DP, Jordan H, Alam S, Gillies MA (2017). The impact of focused echocardiography using the focused intensive care Echo protocol on the management of critically ill patients, and comparison with full echocardiographic studies by BSE-accredited sonographers. J Intensive Care Soc.

[10] Jones AE, Tayal VS, Sullivan DM, Kline JA (2004). Randomized, controlled trial of immediate versus delayed goal-directed ultrasound to identify the cause of nontraumatic hypotension in emergency department patients. Crit Care Med.

[11] Ferrada P, Evans D, Wolfe L, Anand RJ, Vanguri P, Mayglothling J (2014). Findings of a randomized controlled trial using limited transthoracic echocardiogram (LTTE) as a hemodynamic monitoring tool in the trauma bay. J Trauma Acute Care Surg.

[12] Canty DJ, Royse CF, Kilpatrick D, Bowyer A, Royse AG (2012). The impact on cardiac diagnosis and mortality of focused transthoracic echocardiography in hip fracture surgery patients with increased risk of cardiac disease: a retrospective cohort study. Anaesthesia..

[13] Walton-Shirley M (2018). Echocardiography: the good, the bad, and the ugly.

[14] Conlin F, Roy Connelly N, Raghunathan K, Friderici J, Schwabauer A (2016). Focused transthoracic cardiac ultrasound: a survey of training practices. J Cardiothorac Vasc Anesth.

[15] Macdonald MR, Hawkins NM, Balmain S, Dalzell J, McMurray JJV, Petrie MC (2008). Transthoracic echocardiography: a survey of current practice in the UK. Q J Med.

[16] Conlin F, Connelly NR, Eaton MP, Broderick PJ, Friderici J, Adler AC (2017). Perioperative use of focused transthoracic cardiac ultrasound: a survey of current practice and opinion. Anesth Analg.

[17] Via G, Hussain A, Wells M, Reardon R, ElBarbary M, Noble VE (2014). International evidence-based recommendations for focused cardiac ultrasound. J Am Soc Echocardiogr.

[18] Point-of-care ultrasound certificate of completion | Certificate of Completion Program. Am Coll Chest Physicians. [cited 2020 Jun 28]. Available from: https://www.chestnet.org/Education/Advanced-Clinical-Training/Certificate-of-Completion-Program/SHM-COC.

[19] POCUS certificate of completion. [cited 2020 Jun 28]. Available from: https://www.hospitalmedicine.org/clinical-topics/ultrasound/pocus-certificate-of-completion/.

[20] Ultrasound guidelines: emergency, point-of-care, and clinical ultrasound guidelines in medicine. [cited 2020 Jun 29]. Available from: https://www.acep.org/patient-care/policy-statements/ultrasound-guidelines-emergency-point-of%2D%2Dcare-and-clinical-ultrasound-guidelines-in-medicine/.

[21] POCUS practice guidelines. SPOCUS. 2019 [cited 2020 Jun 28]. Available from: https://spocus.org/admin-resources/practice-guidelines/.

[22] Pustavoitau A, Blaivas M, Brown SM, Gutierrez C, Kirkpatrick AW, Kohl BA (2016). Recommendations for achieving and maintaining competence and credentialing in critical care ultrasound with focused cardiac ultrasound and advanced critical care echocardiography.

[23] British Society of Echocardiography. Focused Intensive Care Echocardiography (FICE) accreditation pack. Transthoracic TTE Accreditation. [cited 2020 Oct 12]. Available from: https://www.bsecho.org/Public/Accreditation/Personal-accreditation/Transthoracic%2D%2DTTE-/Public/Accreditation/Accreditation-subpages/Personal-accreditation-subpages/Transthoracic%2D%2DTTE%2D%2Daccreditation.aspx?hkey=a36acc22-8b5c-4ebc-be7e-378ef6d8fc35.

[24] Expert Round Table on Ultrasound in ICU (2011). International expert statement on training standards for critical care ultrasonography. Intensive Care Med.

[25] Ryan T, Berlacher K, Lindner JR, Mankad SV, Rose GA, Wang A (2015). COCATS 4 task force 5: training in echocardiography. J Am Coll Cardiol.

[26] Diaz-Gomez JL, Perez-Protto S, Hargrave J, Builes A, Capdeville M, Festic E (2015). Impact of a focused transthoracic echocardiography training course for rescue applications among anesthesiology and critical care medicine practitioners: a prospective study. J Cardiothorac Vasc Anesth.

[27] Wharton G, Steeds R, Allen J, Phillips H, Jones R, Kanagala P (2015). A minimum dataset for a standard adult transthoracic echocardiogram: a guideline protocol from the British society of echocardiography. Echo Res Pract.

[28] Fletcher SN, Grounds RMIII (2012). Critical care echocardiography: cleared for take up. Br J Anaesth.

[29] European Diploma in advanced critical care EchoCardiography. Eur Soc Intensive Care Med. 2017. Available from: https://www.esicm.org/education/edec-2/.

[30] Pontone G, Moharem-Elgamal S, Maurovich-Horvat P, Gaemperli O, Pugliese F, Westwood M (2018). Training in cardiac computed tomography: EACVI certification process. Eur Heart J Cardiovasc Imaging.

[31] Expert Round Table on Echocardiography in ICU (2014). International consensus statement on training standards for advanced critical care echocardiography. Intensive Care Med.

[32] Kanji HD, McCallum JL, Bhagirath KM, Neitzel AS (2016). Curriculum development and evaluation of a hemodynamic critical care ultrasound: a systematic review of the literature. Crit Care Med.

[33] Higgins J, Thomas J, Chandler J, Cumpston M, Li T, Page M (2019). Cochrane handbook for systematic reviews of interventions version 6.0.

[34] Sterne JA, Hernán MA, Reeves BC, Savović J, Berkman ND, Viswanathan M (2016). ROBINS-I: a tool for assessing risk of bias in non-randomised studies of interventions. BMJ..

[35] Landis JR, Koch GG (1977). The measurement of observer agreement for categorical data. Biometrics..

[36] Alexander JH, Peterson ED, Chen AY, Harding TM, Adams DB, Kisslo JA (2004). Feasibility of point-of-care echocardiography by internal medicine house staff. Am Heart J.

[37] Beraud A-S, Rizk NW, Pearl RG, Liang DH, Patterson AJ (2013). Focused transthoracic echocardiography during critical care medicine training: curriculum implementation and evaluation of proficiency. Crit Care Med.

[38] Caronia J, Kutnick R, Sarzynski A, Panagopoulos G, Mahdavi R, Mina B (2013). Focused transthoracic echocardiography performed and interpreted by medical residents in the critically ill. ICU Dir.

[39] Carrié C, Biais M, Lafitte S, Grenier N, Revel P, Janvier G (2015). Goal-directed ultrasound in emergency medicine: evaluation of a specific training program using an ultrasonic stethoscope. Eur J Emerg Med.

[40] Chisholm CB, Dodge WR, Balise RR, Williams SR, Gharahbaghian L, Beraud A-S (2013). Focused cardiac ultrasound training: how much is enough?. J Emerg Med.

[41] Croft LB, Duvall WL, Goldman ME (2006). A pilot study of the clinical impact of hand-carried cardiac ultrasound in the medical clinic. Echocardiography..

[42] Farsi D, Hajsadeghi S, Hajighanbari MJ, Mofidi M, Hafezimoghadam P, Rezai M (2017). Focused cardiac ultrasound (FOCUS) by emergency medicine residents in patients with suspected cardiovascular diseases. J Ultrasound.

[43] Ferrada P, Anand RJ, Whelan J, Aboutanos MA, Duane T, Malhotra A (2011). Limited transthoracic echocardiogram: so easy any trauma attending can do it. J Trauma.

[44] Gaudet J, Waechter J, McLaughlin K, Ferland A, Godinez T, Bands C (2016). Focused critical care echocardiography: development and evaluation of an image acquisition assessment tool. Crit Care Med.

[45] Hellmann DB, Whiting-O’Keefe Q, Shapiro EP, Martin LD, Martire C, Ziegelstein RC (2005). The rate at which residents learn to use hand-held echocardiography at the bedside. Am J Med.

[46] Johnson BK, Tierney DM, Rosborough TK, Harris KM, Newell MC (2016). Internal medicine point-of-care ultrasound assessment of left ventricular function correlates with formal echocardiography. J Clin Ultrasound.

[47] Labbé V, Ederhy S, Pasquet B, Miguel-Montanes R, Rafat C, Hajage D (2016). Can we improve transthoracic echocardiography training in non-cardiologist residents? Experience of two training programs in the intensive care unit. Ann Intensive Care.

[48] Lucas BP, Candotti C, Margeta B, Evans AT, Mba B, Baru J (2009). Diagnostic accuracy of hospitalist-performed hand-carried ultrasound echocardiography after a brief training program. J Hosp Med.

[49] Manasia AR, Nagaraj HM, Kodali RB, Croft LB, Oropello JM, Kohli-Seth R (2005). Feasibility and potential clinical utility of goal-directed transthoracic echocardiography performed by noncardiologist intensivists using a small hand-carried device (SonoHeart) in critically ill patients. J Cardiothorac Vasc Anesth.

[50] Martin LD, Howell EE, Ziegelstein RC, Martire C, Shapiro EP, Hellmann DB (2007). Hospitalist performance of cardiac hand-carried ultrasound after focused training. Am J Med.

[51] Mjolstad OC, Andersen GN, Dalen H, Graven T, Skjetne K, Kleinau JO (2013). Feasibility and reliability of point-of-care pocket-size echocardiography performed by medical residents. Eur Heart J Cardiovasc Imaging.

[52] Mozzini C, Garbin U, Fratta Pasini AM, Cominacini L (2014). Short training in focused cardiac ultrasound in an internal medicine department: what realistic skill targets could be achieved?. Intern Emerg Med.

[53] Ruddox V, Stokke TM, Edvardsen T, Hjelmesaeth J, Aune E, Baekkevar M (2013). The diagnostic accuracy of pocket-size cardiac ultrasound performed by unselected residents with minimal training. Int J Cardiovasc Imaging.

[54] Ruddox V, Norum IB, Stokke TM, Edvardsen T, Otterstad JE (2017). Focused cardiac ultrasound by unselected residents—the challenges. BMC Med Imaging.

[55] See KC, Ong V, Ng J, Tan RA, Phua J (2014). Basic critical care echocardiography by pulmonary fellows: learning trajectory and prognostic impact using a minimally resourced training model. Crit Care Med.

[56] Smith CJ, Morad A, Balwanz C, Lyden E, Matthias T (2018). Prospective evaluation of cardiac ultrasound performance by general internal medicine physicians during a 6-month faculty development curriculum. Crit Ultrasound J.

[57] Vignon P, Mücke F, Bellec F, Marin B, Croce J, Brouqui T (2011). Basic critical care echocardiography: validation of a curriculum dedicated to noncardiologist residents. Crit Care Med.

[58] Yan BP, Fok JCY, Wong THY, Tse G, Lee APW, Yang XS (2018). Junior medical student performed focused cardiac ultrasound after brief training to detect significant valvular heart disease. IJC Heart Vasc.

[59] Neskovic AN, Edvardsen T, Galderisi M, Garbi M, Gullace G, Jurcut R (2014). Focus cardiac ultrasound: the European Association of Cardiovascular Imaging viewpoint. Eur Heart J Cardiovasc Imaging.

[60] De Geer L, Oscarsson A, Engvall J (2015). Variability in echocardiographic measurements of left ventricular function in septic shock patients. Cardiovasc Ultrasound.

[61] Rajamani A, Shetty K, Parmar J, Huang S, Ng J, Gunawan S (2020). Longitudinal competence programs for basic point-of-care ultrasound in critical care: a systematic review. Chest..

[62] Yamamoto R, Clanton D, Willis RE, Jonas RB, Cestero RF (2018). Rapid decay of transthoracic echocardiography skills at 1 month: a prospective observational study. J Surg Educ.

